# Psychoactive substances belonging to the amphetamine class potently activate brain carbonic anhydrase isoforms VA, VB, VII, and XII

**DOI:** 10.1080/14756366.2017.1375485

**Published:** 2017-09-22

**Authors:** Andrea Angeli, Fabio Vaiano, Francesco Mari, Elisabetta Bertol, Claudiu T. Supuran

**Affiliations:** a Dipartimento Neurofarba, Sezione di Scienze Farmaceutiche e Nutraceutiche, Università degli Studi di Firenze, Florence, Italy;; b Forensic Toxicology Division, Department of Health Sciences, University of Florence, Florence, Italy

**Keywords:** Carbonic anhydrase, activator, amphetamine, methamphetamine, phentermine

## Abstract

Identifying possible new biological activities of psychoactive substances belonging to various chemical classes may lead to a better understanding of their mode of action and side effects. We report here that amines structurally related to amphetamine, a widely used psychoactive substance, such as amphetamine, methamphetamine, phentermine, mephentermine, and chlorphenteramine, potently activate several carbonic anhydrase (CA, EC 4.2.1.1) isoforms involved in important physiological functions. Of the 11 investigated human (h) isoforms, the widespread hCA I and II, the secreted hCA VI, as well as the cytosolic hCA XIII, and membrane-bound hCA IX and XIV were poorly activated by these amines, whereas the extracellular hCA IV, the mitochondrial enzymes hCA VA/VB, the cytosolic hCA VII, and the transmembrane isoform hCA XII were potently activated. Some of these enzymes are abundant in the brain, raising the possibility that some of the cognitive effects of such psychoactive substances might be related to their activation of these enzymes.

## 1. Introduction

**Figure 1.**
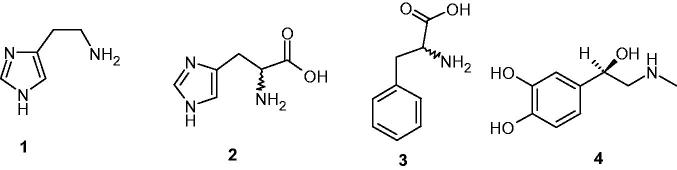
Examples of CAAs: histamine **1**, histidine **2**, phenylalanine **3**, (both L- and D-enantiomers possess this activity), and L-adrenaline **4**.

Drug abuse, especially by young people, constitutes a serious social problem worldwide, with a significant increase in the use/abuse of both “classical” psychoactive substances, such as cocaine, amphetamines, and cannabinoids, as well as new synthetic molecules belonging to a vast array of chemical families, some of which are poorly characterised from the pharmacological and toxicological viewpoints^1–3^. Our interest in this type of compounds is connected to the fact that many of the “classical” drug abuse compounds are primary, secondary, or tertiary amines incorporating the phenethylamine scaffold (such as amphetamine and methamphetamine)^1^^,^^2^. We have showed in earlier works^4–6^ that this type of amines, possessing the general formula Ar–CH_2_CH(R)NHR′, where Ar is an aromatic or heterocyclic ring; R is H, methyl, COOH, or another small compact group (see Figure 1 for some relevant examples), and R′ is H or methyl (with phenethylamine the simplest representative), effectively activate the zinc enzyme carbonic anhydrase (CA, EC 4.2.1.1)^7–10^ involved in a host of physiologic and pathologic processes in organisms all over the phylogenetic tree^11^^,^^12^.

**Figure 2.**
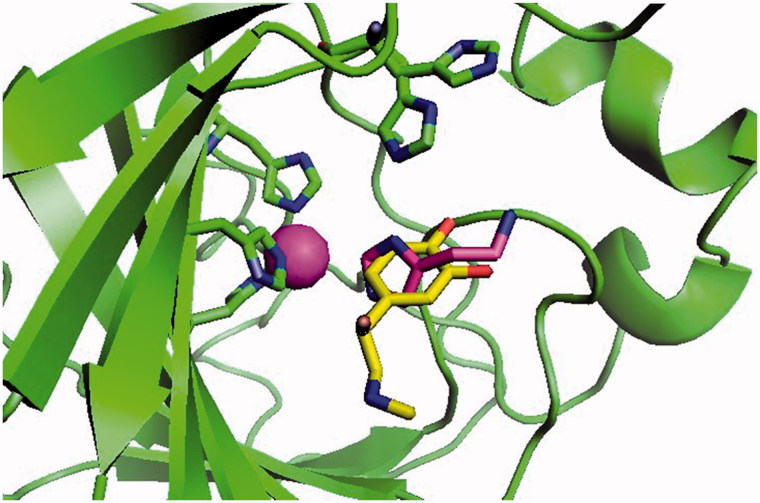
Binding of histamine **1** (magenta, PDB ID: 1AVN)^5^ and L-adrenaline (yellow, PDB ID: 2HKK)^20^**4** to hCA II, as determined by X-ray crystallography. The Zn(II) ion from the enzyme active site is the magenta sphere, with its three His ligands in green (His94, 96, and 119). His64, the natural proton shuttling residues, is shown (in green) in both its “*in*” and “*out*” conformations, above the activator molecules, which are bound at the entrance of the cavity.

CA activators (CAAs) were a controversial issue for a long time: reported in 1941 by three different groups^13–16^, this phenomenon was considered an artefact due to improper experimental measurements for decades^5^^,^^6^, more exactly till 1997, when the first X-ray crystal structure of a CA – activator adduct was reported by some of us^5^. Indeed, histamine **1**, one of the first CAAs investigated^15^ was shown to bind at the entrance of the CA active site cavity (Figure 2) where it participates to the catalytic cycle, more precisely to the rate-determining step of it, which is a proton transfer reaction between a water molecule coordinated to the zinc ion from the enzyme active site to the reaction medium, which generates the zinc hydroxide, nucleophilic species of the enzyme^4–7^. In the catalytic cycle (in the absence of activators), this function is played by the imidazole moiety of a His residue, with a p*K*a around 7, placed in the middle of the active site, which for most human (h) CA isoforms is His64 (hCA I numbering system)^7^. In the presence of CAAs, this proton transfer process is facilitated by the parallel, activator pathway which leads to a facilitated formation of the nucleophilic species of the enzyme^4–7^. Many other X-ray crystal structures of CA – activator adducts have been reported since 1997, such as for example the ones with L-and D-histidine **2**^17^^,^^18^, L- and D-phenylalanine **3**^19^, L-adrenaline **4**^20^, D-Trp^21^, etc. Drug design studies of amine/amino acid based CAAs were also reported^22^^,^^23^. In all these adducts for which the X-ray crystal structure was reported, it has been observed that the activator binds at the entrance of the active site, in a region of the cavity where inhibitors were never observed until 2009, from which they actively participate to the proton shuttling between the active site and the reaction medium^5^^,^^17–21^. In 2009, we have reported the X-ray crystal structure of a coumarin derivative acting as CA inhibitor (CAI) with a totally new mechanism of action^24^. Interestingly, the hydrolysed coumarin, which is the real CAI, binds in the same active site region as the activators, but not having a proton shuttling moiety in its molecule, does not activate but inhibit the enzyme^24^^,^^25^.

As mentioned above, many psychoactive compounds possess the phenethylamine scaffold and the general formula Ar–CH_2_CH(R)NHR′, and they were never investigated as potential CAAs. Here, we report the first such study, showing that amphetamine, methamphetamine, phentermine, mephentermine, and chlorphenteramine, potently activate several CA isoforms, some of which are highly abundant in the brain, where they play important functions connected to cognition and memory, among others^26^^,^^27^.

## 2. Experimental

### 2.1. Chemistry

Compounds **1**–**4** were commercially, highest purity available derivatives from Sigma-Aldrich (Milan, Italy) and were used without further purification. Amines **5**–**9** were certified standards from the standards collection of University of Florence, Struttura di Tossicologia Forense, furnished under authorisation from the Italian Ministry of Health by LGC standards SRL (Milan, Italy).

### 2.2. Carbonic anhydrase assay

A stopped-flow method^28^ has been used for assaying the CA catalysed CO_2_ hydration activity with Phenol red as indicator, working at the absorbance maximum of 557 nm, following the initial rates of the CA-catalysed CO_2_ hydration reaction for 10–100 s. For each activator at least six traces of the initial 5–10% of the reaction have been used for determining the initial velocity. The uncatalysed rates were determined in the same manner and subtracted from the total observed rates. Stock solutions of activator (0.1 mM) were prepared in distilled–deionised water and dilutions up to 0.1 nM were done thereafter with the assay buffer. The activation constant (*K*_A_), defined similarly with the inhibition constant *K*_I_, was obtained by considering the classical Michaelis–Menten equation (Equation (1)), which has been fitted by non-linear least squares by using PRISM 3:

(1) $$v\;=\;v_{\text{max}}/\left\{\text{1 }+\;K_{\text{M}}/\left[S\right]\;\left(\text{1 }+\;{\left[A\right]}_{\text{f}}/K_{\text{A}}\right)\right\}$$

where [*A*]_f_ is the free concentration of activator.

Working at substrate concentrations considerably lower than *K*_M_ ([*S*] ≪ K_M_), and considering that [*A*]_f_ can be represented in the form of the total concentration of the enzyme ([*E*]_t_) and activator ([*A*]_t_), the obtained competitive steady-state equation for determining the activation constant is given by Equation (2)^29–34^:

(2) $$v=v_{0}\cdot K_{\text{A}}/\left\{K_{\text{A}}+({\left[A\right]}_{\text{t}}–\;0.\text{5}\left\{\left({\left[A\right]}_{\text{t}}+{\left[E\right]}_{\text{t}}+K_{\text{A}}\right)\;–\;{\left({\left[A\right]}_{\text{t}}+{\left[E\right]}_{\text{t}}+K_{\text{A}}\right)}^{\text{2}}–\text{ 4}{\left[A\right]}_{\text{t}}.\left[E\right]t{)}^{\text{1}/\text{2}}\right\}\right\}$$

where *v*_0_ represents the initial velocity of the enzyme-catalysed reaction in the absence of activator. All CA isozymes used in the experiments were purified recombinant proteins obtained as reported earlier by our group^29–34^.

## 3. Results and discussion

### 3.1. Chemistry

**Figure 3.**
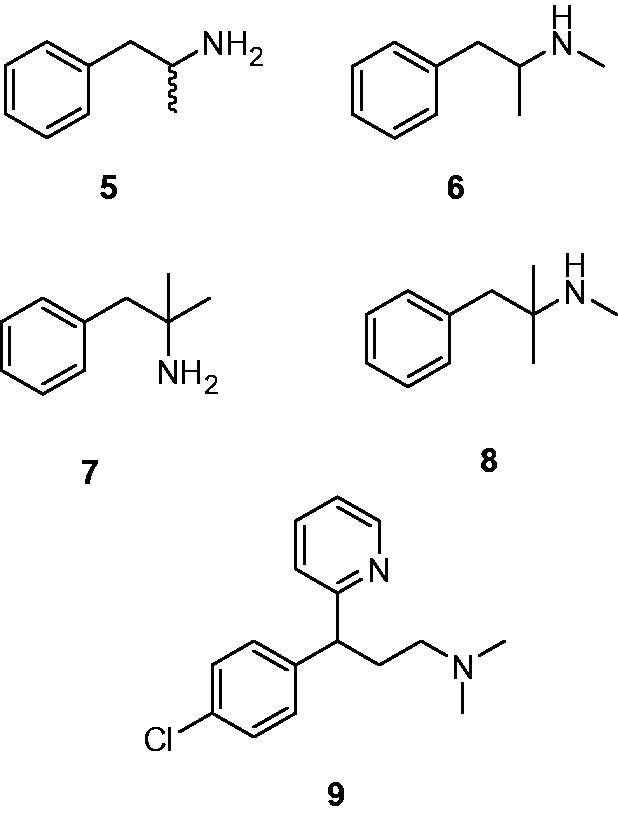
Structure of psychoactive substances investigated as CAAs in the present article: amphetamine **5**, methamphetamine **6**, phentermine **7**, mephentermine **8**, and chlorphenteramine **9**.

Amines **5**–**9** are widely used psychoactive substances (Figure 3). They include primary amines such as amphetamine **5** and phentermine **7**, secondary ones such as methamphetamine **6** and mephentermine **8**, as well as a tertiary amine, chlorphenteramine **9**, which unlike compounds **5**–**8**, which possess the general formula mentioned in the introduction, typical to most CAAs investigated so far^4–6^, incorporates a dimethylamino moiety linked by a three carbon atoms chain to a bulky aromatic moiety of the 4-chlorophenyl type. Furthermore, **9** also incorporates a second bulky moiety, the 2-pyridyl one, making it an interesting candidate to be tested as a CAA, with structural features not encountered in other such derivatives investigated earlier.

### 3.2. CA activation

Amines **5**–**9** are strong central nervous system (CNS) stimulants and were originally used as drugs, for the treatment of attention deficit hyperactivity disorder (ADHD), narcolepsy, obesity, nasal congestion, and depression^35–37^. They interfere with the catecholamine neurotransmitters norepinephrine and dopamine metabolism, by the activation of a trace amine receptor^31^, which leads to an increase of monoamine and excitatory neurotransmitter activity in the brain, leading to emotional and cognitive effects such as euphoria, change in desire for sex, increased wakefulness, accompanied by improved cognitive control^38–40^. However, drug addiction is a serious risk with the recreational use of these substances, with high doses leading to psychosis, such as delusions and paranoia, as well as many other serious side effects^41^.

We investigated the CA activating properties of amines **5**–**9** against 11 catalytically active and physiologically relevant hCA isoforms, that is, the cytosolic hCA I, II, VII, and XIII, the membrane-associated hCA IV, the mitochondrial hCA VA and VB, the secreted hCA VI, as well as the transmembrane isoforms hCA IX, XII, and XIV^7–12^^,^^42^^,^^43^. It should be noted that the tissue distribution and physiological roles of these isoforms are very heterogeneous/complex, but many of them are abundant in the brain^43–45^. Indeed, the human brain as well as the choroid plexus contain a multitude of CA isoforms, although their precise functions are poorly understood^44^. For example, hCA II, the physiological dominant isoform is abundant in the choroid plexus, oligodendrocytes, myelinated tracts, astrocytes, and myelin sheaths in the vertebrates brain^44^. The membrane-associated CA IV is located on the luminal surface of cerebral capillaries, and associated with the blood–brain barrier, being also concentrated in layers III and VI within the cortex, hippocampus, and thalamus of all investigated mammals^44^^,^^45^. Other studies demonstrated the presence of the mitochondrial CA VA in the nervous tissues, where the enzyme is expressed in astrocytes and in neurons, being probably involved in biosynthetic processes such as neoglucogenesis, lipogenesis, etc^36^^,^^37^. The expression of CA VII is of particular interest, as relatively high levels have been observed throughout the cortex, hippocampus and thalamus (CA VII also shows a very good catalytic activity for the physiological reaction)^44^ and is probably the target for CA inhibitors acting as anti-neuropathic pain and anti-epileptic agents^44^^,^^45^. CA IX was shown to be overexpressed in many neurologic cancers such as glioma, ependymoma, hemangioblastoma, meningioma as well as choroid plexus tumors^42^. CA XII is also connected to cancers and has the same expression pattern as CA IX in brain tumors^42–44^. However, CA XII is also present in normal tissues and a high level of this isoform was reported in the choroid plexus^42^. CA XIV is expressed in nuclei and nerve tracts associated with pontine, medullary, and hippocampal functions^43^. CA XIV was also shown to be located on the plasma membrane of some neurons and on axons of both mouse and human brain^43^^,^^44^. With so many CA isoforms in the CNS it is rather difficult to ascertain their precise physiological role, but some indirect information may be drawn from the fact that positively-charged, membrane-impermeant CAAs (or CAIs) do not show central nervous system effects when administered intra-peritoneally, which may be explained by the fact that intracellular (cytosolic or mitochondrial) CA isoforms are involved in the activation/inhibition phenomena. As mentioned briefly above, CA activation in the brain leads to enhanced memory, through the activated extracellular signal-regulated kinase (ERK) pathway, which is involved in a critical step for memory formation in the cortex and the hippocampus, two brain areas known to play a crucial role in memory processing, as recently demonstrated by one of our groups^27^.

CA activation data against these 11 isoforms with psychotropic amines **5**–**9** and standard CAAs **1**–**4** are shown in Tables 1 and 2.

**Table 1.** CA activation of isoforms hCA I, II, VII, and XIII (cytosolic) and IV (membrane-associated) with compounds **1**–**9**, by a stopped-flow CO_2_ hydrase assay^28^.

	*K*_A_^a^ (µM)				
Compound	hCA I	hCA II	hCA IV	hCA VII	hCA XIII
**1**	2.1	125	25.3	37.5	4.7
**2** (L-His)	0.03	0.90	7.3	0.92	0.13
**3** (L-Phe)	0.07	0.013	36.3	10.9	1.02
**4**	0.09	96	45	60	nt
**5**	>150	>150	0.094	0.91	24.1
**6**	>150	>150	0.051	0.93	25.6
**7**	>150	>150	0.074	0.89	54.2
**8**	>150	>150	1.03	0.64	48.3
**9**	>150	>150	0.055	0.098	79.5

nt: not tested. Data for **1**–**4** from Refs.^17–21^^,^^30–34^.

a Errors in the range of ±5–10% of the reported values (data not shown) from three different assays.

**Table 2.** CA activation of isoforms hCA VA, VB (mitochondrial), VI (secreted), and IX, XII, and XIV (trans-membrane) with compounds **1**–**9**, by a stopped-flow CO_2_ hydrase assay^28^.

	*K*_A_^a^ (µM)					
Compound	hCA VA	hCA VB	hCA VI	hCA IX	hCA XII	hCA XIV
**1**	0.010	3.52	6.50	35.1	27.9	0.010
**2** (L-His)	1.34	0.97	32.0	9.71	37.5	0.90
**3** (L-Phe)	9.81	10.45	1.23	16.3	1.38	0.24
**4**	63	67	nt	0.87	0.41	36.1
**5**	0.81	2.56	>150	>150	0.64	9.15
**6**	0.92	0.78	>150	>150	0.80	7.38
**7**	0.53	0.62	>150	34.6	3.24	12.7
**8**	0.37	0.24	>150	25.8	6.12	18.1
**9**	0.31	0.75	>150	34.1	0.97	6.81

nt: not tested. Data for **1**–**4** from Refs.^17–21^^,^^30–34^.

a Errors in the range of ±5–10% of the reported values (data not shown) from three different assays.

The following structure–activity relationship (SAR) for the activation of these enzymes with psychotropic amines **5**–**9** can be drawn from data of Tables 1 and 2:

- The cytosolic, widespread isoforms hCA I and II were not significantly activated by these amines up to concentrations as high as 150 µM, whereas hCA XIII, another cytosolic isoform with a more particular expression pattern (as it is widespread in many organs but with quite low expression level)^42^ is moderately activated by amines **5**–**9**, with activation constants in the range of 24.1–79.5 µM. The best activators of hCA XIII were amphetamine **5** and methamphetamine **6**, whereas phentermine **7**, mephentermine **8**, and chlorphenteramine **9** showed a decreased potency. It is interesting to note the differences between the activating effects of amines **5**–**9** and the standard CAAs **1**–**4**: hCA I for example is effectively activated by compounds **1**–**4**, whereas hCA II is poorly activated by histamine 1 and L-adrenaline **4**, but highly effectively activated by amino acids such as L-His and D-Phe (Table 1). hCA XIII is also rather well activated by **1**–**3** (L-adrenaline’s effects on this isoform were not investigated).
- hCA VII, the brain-associated cytosolic CA isoform^46^, known to be involved in the antiepileptic and antineuropathic pain effects of the CAIs^44^^,^^45^, was effectively activated by the psychotropic amines investigated here, with *K*_A_s ranging between 98 nM and 0.93 µM. The most effective hCA VII activator was chlorphenteramine **9** whereas the remaining derivatives, possessing the phenethylamine scaffold showed rather similar, submicromolar activation constants (*K*_A_s of 0.64–0.93 µM). No major differences in the activating capacity were observed between the primary and secondary amines, or between the derivatives possessing the relatively not sterically hindered α-methyl group near the amino functionality, such as **5** and **6**, compared to the sterically hindered, structurally related amines **7** and **8** (Table 1). It should also be noted that except L-His, which has the same level of activity as the psychotropic amines investigated here, the other standard CAAs, such as **1**, **3**, and **4**, were quite ineffective as hCA VII activators (Table 1).
- The membrane-associated, rather widespread (in endothelial cells of blood capillaries in many organs such as lungs, gastro-intestinal tract, brain, epithelial cells of gallbladder, renal tubules, reproductive organs, etc.)^43^ isoform hCA IV was the most sensitive one to activation by amines **5**–**9**, which showed *K*_A_s ranging between 51 nM and 1.03 µM. The best hCA IV activator was methamphetamine **6** and chlorphenteramine **9** (*K*_A_s of 51–55 nM), followed by phentermine **7** and amphetamine **5** (*K*_A_s of 74–94 nM). The sterically hindered (at the amino moiety) mephentermine **8** was the least effective CAA with a *K*_A_ of 1.03 µM. There is a huge difference of activity between the psychotropic amines investigated here **5**–**9**, which act as efficient or highly efficient hCA IV activators, and compounds **1**–**4**, which were quite ineffective such agents with *K*_A_s in the range of 7.3–45 µM (Table 1).
- The two mitochondrial isoforms hCA VA and VB, involved in many metabolic reactions (ureagenesis, lipogenesis, neoglucogenesis, etc.)^47^ and also present in the brain^43^, were also effectively activated by amines **5**–**9**, with *K*_A_s ranging between 0.24 and 2.56 µM (Table 2). The best activators against these isoforms were mephentermine **8** against hCA VB (*K*_A_ of 240 nM) and chlorphenteramine **9** against hCA VA (*K*_A_ of 310 nM), with the remaining compounds (except amphetamine **5** against hCA VB which showed a *K*_A_ of 2.56 µM) showing a rather effective, submicromolar activation profile against both isoforms and with a flat SAR (*K*_A_s ranging in a narrow interval of 0.37–0.92 µM, Table 2). It should be noted that apart histamine **1**, which is an effective hCA VA activator (*K*_A_ of 10 nM), the standard CAAs **2**–**4** were generally less effective mitochondrial CA activators compared to the psychotropic amines **5**–**9** (Table 2).
- Similar to hCA I and II, the secreted isoform hCA VI (present in the saliva and milk)^30^, was not significantly activated by amines **5**–**9** up until 150 µM concentration of activator, whereas compounds **1**–**3** showed a much better activating efficacy (Table 2).
- The tumor-associated, transmembrane isoform hCA IX was not significantly activated by amphetamine **5** and methamphetamine **6** (*K*_A_s >150 µM), whereas the remaining psychotropic amines **7**–**9** showed weak activating effects, with *K*_A_s of 25.8–34.6 µM. However, the second tumour-associated, transmembrane isoform hCA XII showed a very different activation profile with these compounds compared to hCA IX. Indeed, amphetamine **5**, methamphetamine **6**, and chlorphenteramine **9** were submicromolar activators (*K*_A_s of 0.64–0.97 µM) whereas the sterically hindered amines **7** and **8** were one order of magnitude less efficient as hCA XII activators (*K*_A_s of 3.24–6.12 µM). The primary amines **5** and **7** were in this case more effective activators compared to the corresponding secondary amines **6** and **8**. Among the standard CAAs, only L-adrenaline **4** showed the same level of activity as amines **5** and **6**, the remaining derivatives **1**–**3** being generally less effective as hCA XII activators (Table 2).
- hCA XIV, another transmembrane isoform not connected with tumours, and present in neurons, hepatocytes, renal tubules^42^^,^^43^, was moderately activated by amines **5**–**9**, which showed *K*_A_s of 6.81–18.1 µM. The best hCA XIV activator was chlorphenteramine **9** whereas the least effective one was mephentermine **8**. Some of the standard CAAs, such as histamine **1** showed much more effective, low nanomolar activating effects against this isoform.

## 4. Conclusions

We investigated psychotropic amines based on the phenethylamine scaffold, such as amphetamine **5**, methamphetamine **6**, phentermine **7**, mephentermine **8**, and the structurally diverse chlorphenteramine **9**, for their activating effects on 11 CA isoforms of human origin, hCA I, II, VII, and XIII (cytosolic isoforms), the membrane-associated hCA IV, the mitochondrial hCA VA, and VB, the secreted hCA VI, as well as the transmembrane isoforms hCA IX, XII, and XIV. The widespread hCA I and II, the secreted hCA VI, as well as the cytosolic hCA XIII and membrane-bound hCA IX and XIV were poorly activated by these amines, whereas the extracellular hCA IV, the mitochondrial enzymes hCA VA/VB, the cytosolic hCA VII, and the transmembrane isoform hCA XII were potently activated. Some of these enzymes (hCA VII, VA, VB, XII) are abundant in the brain, raising the possibility that some of the cognitive effects of such psychoactive substances might be related to the activation of these enzymes. Furthermore, unlike the CAIs which are in clinical use for decades for a multitude of applications^47a^^,^^48–61^, the CAAs started to be considered only recently for possible pharmacologic applications in memory/cognition therapy^27^. This work may bring new lights on the intricate relationship between CA activation by this type of compounds and the multitude of pharmacologic actions that they can elicit.
